# Current Status of Breast Reconstruction in Southern China: A 15 Year, Single Institutional Experience of 20,551 Breast Cancer Patients

**DOI:** 10.1097/MD.0000000000001399

**Published:** 2015-08-28

**Authors:** Chen Jia-jian, Huang Nai-si, Xue Jing-yan, Yang Ben-long, Liu Guang-yu, Di Gen-hong, Shao Zhi-min, Wu Jiong

**Affiliations:** From the Department of Breast Surgery, Fudan University Shanghai Cancer Center; and Department of Oncology, Shanghai Medical College, Fudan University, Shanghai, China (CJJ, HNS, XJY, YBL, LGY, DGH, SZM, WJ).

## Abstract

The study of this study is to assess the current status and trend of the application of breast reconstruction in China.

A retrospective review of all patients who had received surgical treatment for breast cancer in the Fudan University Shanghai Cancer Center between January 1999 and June 2014 was performed. The clinicopathological and epidemiological parameters and the follow-up information of each patient were collected.

A total of 20,551 patients with 20,974 surgeries were identified. Of those, the rates of patients received mastectomy, breast conserving therapy, and breast reconstruction were 81.2% (17,040 cases), 15.3% (3216 cases), and 3.4% (718 cases), respectively. Skin-sparing mastectomy with autologous breast reconstruction was algate the dominant option for breast reconstruction although a rapid growth in the application of prosthetic reconstructions was observed in recent years. The rates of complications that required reoperation in patients reconstructed with latissimus dorsi myocutaneous flap, pedicled transverse rectus abdominis myocutaneous flap, free flaps, and prosthesis were 1.2%, 8.5%, 11.4%, and 10.5%, respectively, while the revision rates were 0.7%, 6.1 %, 5.3%, and 2.3%, respectively. Multiple regression analysis confirmed that types of surgery did not affect the disease-free survival of breast cancer patients.

Skin-sparing mastectomy with breast reconstruction is oncologically safe while achieving satisfactory aesthetic outcomes. Autologous reconstruction remains the most commonly used technique while there is a rapid increase of prosthetic reconstruction in recent years. The low demand for breast aesthetics among Chinese women, defects of healthcare system, and the limited availability of recourses impeded the development of breast reconstruction techniques in China.

## INTRODUCTION

The surgical management of breast cancer has experienced substantial directional revolution, from radical to minimal surgery,^1^ which reflects the further understanding of the disease from anatomical to the extension of biological, and the more consideration about the quality of life and cosmetic appearance.

However, total mastectomy remains the dominant option of surgical management for breast cancer in China.^2,3^ The reasons are thought be the smaller size of breasts in Chinese women and the deep-rooted traditional concepts toward cancer among Chinese patients, despite the fact that a growing proportion of patients can be diagnosed at much earlier stages owing to the increasing public awareness and the development of screening programs.

In recent years, attentions have been focused on oncoplastic breast surgery (OBS), an amalgamation of oncological surgical techniques and plastic reconstructive techniques to produce a significantly improved aesthetic outcome for the breast cancer patients, while not affecting the multidisciplinary adjuvant treatment.^4^ As a result, there have been increasing demands by patients and surgeons alike for skin-sparing mastectomy (SSM) and nipple-areola sparing mastectomy, combined with immediate breast reconstruction.^5,6^

Fudan University Shanghai Cancer Center is one of the major cancer centers in China with over 3000 new surgical cases for breast cancer per year in recent years. The present study aims to assess the development and current status of breast reconstruction in a single institution.

## MATERIALS AND METHODS

### Patients

A retrospective review of all patients who had been surgically treated for breast cancer in the Fudan University Shanghai Cancer Center between January 1999 and June 2014 was performed, and a total of 20,551 breast cancer patients with 20,974 surgeries were included in the present study. The exclusion criteria including: male patients, stage IV patients who had received palliative operations. The clinicopathological and epidemiological parameters and follow-up information were available in the electronic medical records after 2004. For patients treated prior to 2004, the scanned medical records were reviewed, in which some pathological data were missing, especially for the patients received biopsy elsewhere. Complications and revisions for patients received breast reconstruction were also recorded. Pre- and postoperative photographs were taken for patients receiving breast reconstruction. An informed consent for the academic use of photographs was obtained from each patient. The medical data from patients treated between January 2004 and December 2012 were applied for the analysis of survival distributions. This study was approved by the Fudan University Shanghai Cancer Center ethics committee.

### Methods of Breast Reconstruction

For patients planned for immediate autologous or prosthetic breast reconstruction, standard SSM was performed. Autologous breast reconstructions included latissimus dorsi myocutaneous flaps (LDMF) with or without implants, pedical transverse rectus abdominis myocutaneous (pTRAM) flaps, and free tissue transfers using abdominal flaps including free TRAM, muscle-sparing TRAM, and deep inferior epigastic perforator flap.^7^ Prosthetic reconstructions included tissue expander-implant placement and implant placement alone.

For a more accurate analysis of postoperative complications, only those requiring reoperation were included.

### Measurement of Workload Load

To further explore the possible reasons for the paradigm shift of the surgical approaches, the workload per surgeon was introduced in the analysis. Based primarily on the length and complexity of surgery, a scoring system was created. The scores for each breast conservation therapy, total mastectomy, prosthetic breast reconstruction, and autologous reconstruction were set as 1.0, 1.5, 2.0, and 4.0, respectively. The total scores per year for each surgeon reflected the amount of the surgeon's workload.

### Statistical Analysis

The independent samples *t*-test and analysis of variance were performed to compare continuous variables, while Fisher exact test and Pearson Chi-square test were used to analyze categorical variables. Multivariable regression analysis was performed to determine independent risk factors for breast cancer-specific disease-free survival. Survival distributions were analyzed using the Kaplan–Meier method with the data from patients treated between January 2004 and December 2012.

Statistical analyses were performed using SPSS version 20.0 (SPSS Inc., Chicago, IL). A *P* value of less than 0.05 was considered statistically significant.

## RESULTS

Between January 1999 and June 2014, a total of 20,551 patients with breast cancer received surgical treatment at the Department of Breast Surgery, Fudan University Shanghai Cancer Center. Among these, 470 patients had synchronous or metachronous bilateral breast malignancies and received surgeries for both breasts. Forty seven patients with metachronous bilateral breast cancers, however, received their first surgeries before 1999. These 47 procedures were not included in the database. Therefore, a total of 20,551 breast cancer patients with 20,974 surgeries were included in the present study.

### Distribution and Trends of Surgical Approaches

**FIGURE 1:**
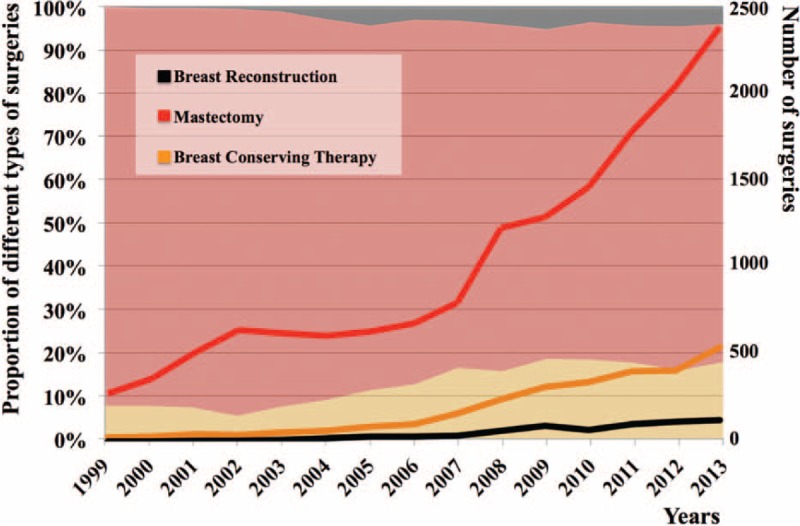
Distribution of breast surgeries between January 1999 and December 2013. In the square background, the gray, red, and yellow zones represent the changes of the percentage of oncoplastic surgeries, mastectomy, and breast conservation therapy, respectively. The lines in different colors indicate the number of surgeries performed.

The number of cases increased rapidly in recent years (Figure 1). Of the 20,974 surgeries for breast cancer, there were 17,040 cases of total mastectomy (81.2%) with an average annual growth rate of 16.7%. Breast conservation therapy accounted for 15.3% (3216 cases). Although there was a steady increase of reconstructive cases, the total number remained small, only 718 cases or 3.4% of all surgical cases.

Table 1 shows the demographic and clinicopathological characteristics of patients who had undergone total mastectomy, breast conservation therapy, and OBS, respectively. Patients in the breast reconstruction group were significantly younger than those with total mastectomy or breast conservation therapy (*P* < 0.001), and had a much higher proportion of in situ diseases (*P* < 0.001) and Her-2/neu positive diseases (*P* < 0.001). The mean length of hospital stay in the breast reconstruction group was 1 day longer than that in the total mastectomy group (*P* = 0.006) and 7 days longer than those in the lumpectomy group (*P* < 0.001).

**TABLE 1:**
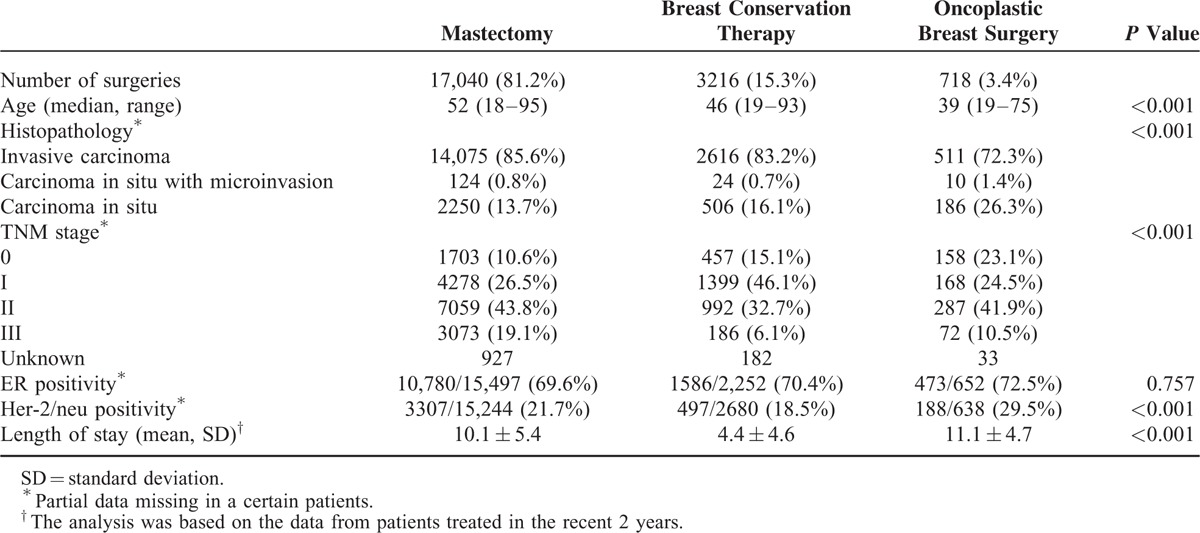
Patient Demographic and Clinicopathological Characteristics

### Immediate and Delayed Breast Reconstruction

A total of 697 patients received breast reconstruction, among whom, 1 patient with metachronous bilateral breast cancer received bilateral breast reconstruction at the time of the second mastectomy, 1 year after the first. A total of 670 (96.1%) patients with 687 procedures received immediate breast reconstruction. The other 28 (4.0%) patients with 31 procedures received delayed breast reconstruction.

**FIGURE 2:**
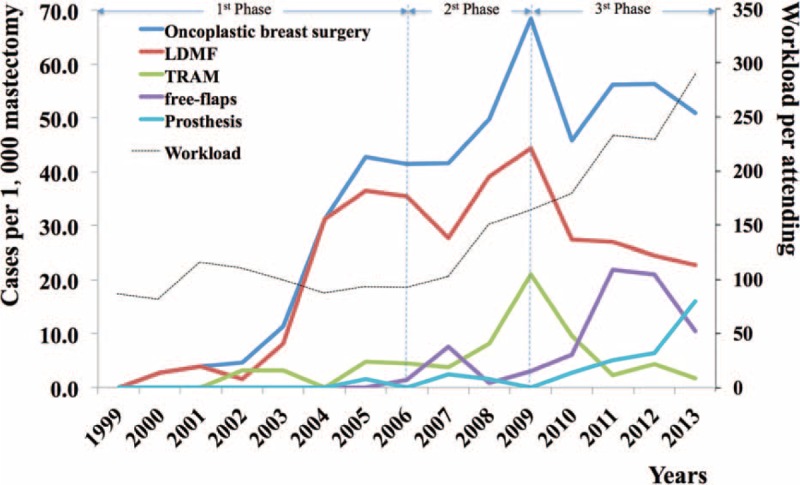
Changes of breast reconstruction techniques overtime and workload per surgeon per year. The black dotted line indicates the increasing workload in our center. The vertical lines separate the 3 different phages of the reconstruction paradigms.

For the patients who received SSM with immediate breast reconstruction, LDMF with or without implants was the most common reconstructive procedure (418 cases, 60.8%). The free-flap breast reconstruction was performed in 132 (19.2%) cases, with a trend of increase overtime. The pTRAM flap was used in 82 cases (11.9%) and was popular between 2008 and 2010 but subsequently decreased with the increased application of free flaps. The remaining 86 (12.5%) cases received prosthetic breast reconstruction, including tissue expander-implant placement (33 cases) or implant alone (53 cases). Prosthetic breast reconstruction started late in China but showed a trend of rapid increase in recent years (Figure 2).

Patients with delayed breast reconstruction had a median time interval of 24.3 months after mastectomy. Free flap reconstruction was most common for this group of patients (67.7%), followed by the pedicled TRAM flaps (22.6%), the LDMF with implant (6.5%), and implant only (3.2%).

### Autologous-Based and Implant-Based Immediate Breast Reconstruction

Among the patients who did not fit the criteria for breast conservation therapy, there appeared to be a rising trend in the application of breast reconstruction (Figure 2). A total of 632 patients received autologous-based reconstruction and 86 patients received implant-based breast reconstruction.

No significant difference was found in patient's age between immediate prosthetic reconstructions and autologous-based reconstructions (37.1 vs 39.1, *P* = 0.070), although the age differences between abdominal flap and nonabdominal flap reconstruction patients were significant (42.6 vs 37.4, *P* < 0.001). The body mass index of patients with prosthetic breast reconstructions was significantly lower than that of patients with autologous-based breast reconstruction (20.6 vs 21.7, *P* = 0.002).

The length of hospital stay for implant-based reconstruction was significantly shorter than that of autologous-based reconstruction (7 vs 13 days, *P* < 0.001).

There appeared to be 3 different phases in the reconstruction paradigm, accompanying with the rising workload (Figure 2). From 2001 to 2006, the workloads of surgeons were similar by year. Breast reconstruction had just become increasingly popular in our center. During that time period, the types of reconstruction were largely limited to LDMF with or without implant placement. From 2006 to 2009, the pTRAM flap was introduced, which replaced the LDMF as the first choice for breast reconstruction, especially in those with larger breasts. After 2009, free deep inferior epigastic perforator flaps became popular and largely replaced pTRAM flap reconstruction. Meanwhile, with the dramatic increase of surgeon's workload, there was a clear trend of increasing prosthetic reconstructions with a slight decrease of autologous reconstructions.

### Complication and Revision Rates

The rates of complications requiring reoperation were 1.2%, 8.5%, 11.4%, and 10.5%, for patients with LDMF, pTRAM flap, free-flap, and prosthetic reconstructions, respectively. Breast revision rates were 0.7%, 6.1%, 5.3%, and 2.3%, respectively. The complications following prosthetic reconstruction included infection (3 cases), rupture or leakage of the tissue expander (4 cases), postoperative bleeding (1 case), and removing the implant on account of discomfort and pain (1 case). Total flap loss following free-flap reconstruction was 2.3% (3 cases) with a reexploration rate of 6.1% (8 cases). There were also 2 cases (1.5%) of fat necrosis requiring surgical intervention. There were no statistically significant differences in complication and revision rates between immediate and delayed breast reconstructions on the same type of surgery. Mastopexy for the contralateral breasts was performed in 1.5%, 3.7%, 3.9%, and 8.7% of patients with LDMF, pTRAM flap, free-flap, and prosthesis, respectively.

### Nipple-Areolar Reconstruction and Tattooing

Only 92 (13.2%) patients received nipple reconstruction. The nipple reconstruction rates were 8.9%, 11.0%, 32.6%, and 8.1% for patients with LDMF, TRAM flap, free-flap, and prosthetic reconstructions, respectively. The median time interval between breast reconstruction and nipple reconstruction was 8.9 months, ranging from 0.2 to 61.6 months. Patients undergoing delayed breast reconstruction had a significantly higher nipple reconstruction rate (32.3%) than those with immediate breast reconstruction (12.8%, *P* = 0.023). Patients who had undergone bilateral breast reconstructions also had a higher nipple reconstruction rate than unilateral ones (33.3% vs 8.3%, *P* = 0.003).

### Oncological Safety of Oncoplastic Breast Surgery

Survival information was available on 12,625 patients treated between January 2004 and December 2012. The median follow-up was 37.1 months. Overall breast cancer-specific disease-free survival rates were similar between the mastectomy groups (95.8% vs 92.8%, *P* = 0.355), and between the OBS and the breast conservation groups (95.8% vs 95.6%, *P* = 0.162). The disease-free survival of patients who had undergone breast conservation therapy was understandably better than those of mastectomy (*P* < 0.001). Multiple regression analysis further confirmed that types of surgeries did not affect the breast cancer-specific disease-free survival, while the Tumor Node Metastasis

stage and ER status were independent risk factors (Table 2).

**TABLE 2:**
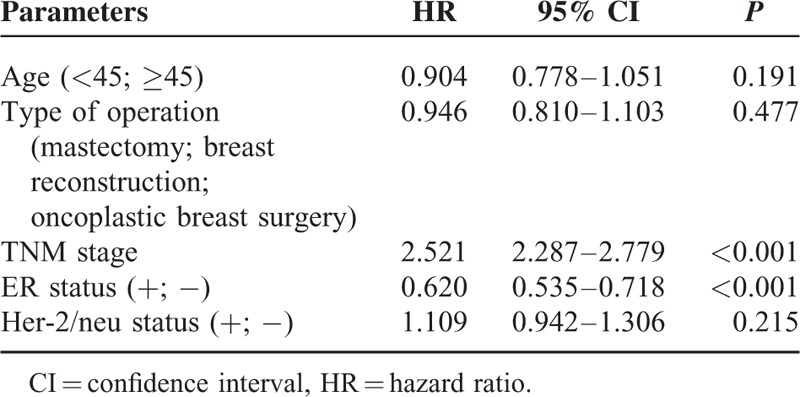
Multiple Regression Analysis of Risk Factors for Breast Cancer-Specific Disease-Free Survival

## DISCUSSION

Our center treats patients from all over China, especially from southern China. Therefore, this large study may well represent the current status of breast reconstruction in southern China.

### Possible Reasons for the Limited Rate of Breast Reconstruction

The rate of total mastectomy was significantly higher in China than in European and American countries.^8,9^ One of the reasons is thought to be the smaller breast volume of Chinese women, which is less amenable for breast conservation therapy.^10,11^ In addition, the deep-rooted traditional concepts toward cancer, a firm belief that all cancers should be radically resected, may also prompted a portion of breast cancer patients who would otherwise be suitable for breast conservation therapy to refuse the appropriate surgery. Lastly, a shortage of medical resources, radiation therapy, which needed to be given as part of breast conservation therapy, may also contribute to the high rate of mastectomy in China. However, the high rate of total mastectomy did not translate to a high rate of breast reconstruction. Data from the past 15 years showed a rather stable low reconstruction rate at about 3.5%.^8,9,12,13^ The main reason is thought to be that traditional Chinese women have low demand for their body image, and many of them are unaware of the possibility of breast reconstruction. This is particularly true for the older generations in China, which explains the 10-year age difference between patients with and without breast reconstruction.^14^ The low rate of nipple reconstruction, only 13.2%, also reflects this trend. Most patients were satisfied with their appearance without nipple and areolar reconstruction.

The availability and distribution of medical recourses may also contribute to the low reconstruction rate. In China, most cancer hospitals do not have a plastic surgery unit. Therefore, all the OBS procedures are done by the breast surgeons, generating tremendous amount of workload. For instance, the number of breast surgeons in our department increased from 13 in 2006 to 15 in 2014. However, there was a 4-fold increase of the number of surgeries performed for breast cancer in the same time period (Figure 1). The heavy workload hampered the generalized application of reconstruction techniques and protracted the accomplishment of learning for the reconstruction techniques, especially for microvascular skills.

Several other factors that have been reported to influence the overall rates of breast reconstruction elsewhere may have little effect in Chinese patients. For example, privately insured patients were reported to be more likely to receive reconstruction,^15,16^ but this is not a major issue in China because the total cost for SSM and immediate free-flap breast reconstruction is merely ¥5197 ($840). It has also been reported that a dramatic increase of bilateral mastectomies, performed as either a contralateral prophylactic mastectomy or a bilateral prophylactic mastectomy,^17,18^ in Western countries changed the reconstruction rates. However, this has little effect in China because such procedures are rarely performed.

### Shift in Breast Reconstruction Paradigm

Recent studies in the United States have shown a dramatic increase in prosthetic breast reconstruction from 40% to 74%^12,18,19^ partly due to the changed mastectomy patterns – a 15% and 12% per year increased application of contralateral and bilateral prophylactic mastectomies.^18^ However, this is not the case in China for multiple reasons, such as the lack of public awareness, scientific guidelines, and the feasibility of tests for breast cancer; 1/2 gene mutation, as well as cultural differences. Therefore, autologous breast reconstruction remains the popular option (Figure 2). Nevertheless, there is a clear trend of increased implant-based reconstructions in recent years as shown in Figure 2. Autologous breast reconstructions are complex and resource intensive.^8^ Surgeons have to take the operative time, hospital admission, length of stay, and the number of patients waiting for surgery into consideration. In addition, the incentive to perform combined SSM and breast reconstruction with pedicle flaps (¥4438 or $715), free flaps (¥5197 or $840), or prosthesis (¥3254 or $525) is not very appealing compared to mastectomy only (¥1150 or $185). This is particularly discouraging for free-flap reconstruction.

### Complications and Oncological Safety of Breast Reconstruction

To avoid the possibility of missing documentation of complications, only complications that required operative intervention were included in this study. The abdominal-based flaps had a significantly higher complication rate or revision rate compared with LDMF. However, the complication rate of prosthetic breast reconstruction was surprizingly high, even higher than the free-flap group. This is, however, most likely the result of learning curve. Implant-based reconstruction is just becoming popular in China.

The oncologic safety of SSM and breast reconstruction has been well documented in the past decades.^7,20,21^ In this study, types of surgery did not appear to be an independent factor for breast-specific disease-free survival.

## CONCLUSION

The present study provided an overview of the current status of breast reconstruction in China. The low demand for aesthetic outcomes in Chinese women defects in the healthcare system, and the availability of recourses impeded the development of OBS in China, although there appeared to be a trend of increasing demand for breast reconstruction. Autologous breast reconstruction remains the dominant procedure, especially for delayed breast reconstructions, although a sharp increase of prosthetic reconstruction was observed in the last 2 or 3 years.
